# Comparison of the dosimetric accuracy of proton breast treatment plans delivered with SGRT and CBCT setups

**DOI:** 10.1002/acm2.13357

**Published:** 2021-07-20

**Authors:** Michael J. MacFarlane, Kai Jiang, Michelle Mundis, Elizabeth Nichols, Arun Gopal, Shifeng Chen, Nrusingh C. Biswal

**Affiliations:** ^1^ Department of Radiation Oncology University of Maryland School of Medicine Baltimore MD 21201 USA

## Abstract

**Purpose:**

To compare the dosimetric accuracy of surface‐guided radiation therapy (SGRT) and cone‐beam computed tomography (CBCT) setups in proton breast treatment plans.

**Methods:**

Data from 30 patients were retrospectively analyzed in this IRB‐approved study. Patients were prescribed 4256–5040 cGy in 16–28 fractions. CBCT and AlignRT (SGRT; Vision RT Ltd.) were used for treatment setup during the first three fractions, then daily AlignRT and weekly CBCT thereafter. Each patient underwent a quality assurance CT (QA‐CT) scan midway through the treatment course to assess anatomical and dosimetric changes. To emulate the SGRT and CBCT setups during treatment, the planning CT and QA‐CT images were registered in two ways: (1) by registering the volume within the CTs covered by the CBCT field of view; and (2) by contouring and registering the surface surveyed by the AlignRT system. The original plan was copied onto these two datasets and the dose was recalculated. The clinical treatment volume (CTV): V_95%_; heart: V_25Gy_, V_15Gy_, and mean dose; and ipsilateral lung: V_20Gy_, V_10Gy_, and V_5Gy_, were recorded. Multi and univariate analyses of variance were performed to assess the differences in dose metric values between the planning CT and the SGRT and CBCT setups.

**Results:**

The CTV V_95%_ and lung V_20Gy_, V_10Gy_, and V_5Gy_ dose metrics were all significantly (*p* < 0.01) lower on the QA‐CT in both the CBCT and SGRT setup. The differences were not clinically significant and were, on average, 1.4–1.6% lower for CTV V_95%_ and 1.8%–6.0% lower for the lung dose metrics. When comparing the lung and CTV V_95%_ dose metrics between the CBCT and SGRT setups, no significant difference was observed. This indicates that the SGRT setup provides similar dosimetric accuracy as CBCT.

**Conclusion:**

This study supports the daily use of SGRT systems for the accurate dose delivery of proton breast treatment plans.

## 1 INTRODUCTION

Imaging is indispensable for the accurate planning and delivery of modern radiation therapy, especially when the target volume is not at or near the surface and therefore cannot be localized for treatment by visual inspection alone. In‐room and on‐board image‐guided radiation therapy (IGRT) systems (planar, volumetric, video‐, or ultrasound‐based) are used to obtain temporal information about the target position and motion (within the same session or between consecutive sessions), compare it with reference imaging, and provide feedback on correcting the patient setup and optimizing target localization.^1^ Surface‐guided radiation therapy (SGRT) systems have recently grown in popularity for certain disease sites because they are easy to setup and capable of real‐time monitoring the patient without the radiation burden associated with radiographic imaging or requiring tattoos or markers.^2^, ^3^, ^4^, ^5^

SGRT systems like AlignRT (Vision RT Ltd.), Identify (Varian Medical Systems, Inc.), and others work by projecting a known light field pattern onto the patient and monitoring this projection with a network of video cameras distributed around the room.^2^, ^3^, ^6^ The SGRT software extracts spatial features of the light field pattern from each video camera feed and, using mono‐ or stereoscopic imaging techniques, reconstructs a 3D rendering of the patient surface in real‐time. The software then compares the reconstructed surface with a reference surface—such as the patient surface in the planning CT or a surface acquired earlier with the SGRT system—to provide suggestions on how to improve patient setup accuracy. It can also alert the therapist or halt beam delivery if there is any substantial patient motion during treatment delivery or provide beam gating and patient feedback for respiratory motion management.

Several studies have evaluated the setup accuracy of SGRT systems compared to commonplace marker and imaging‐based setup techniques for breast and chest wall irradiation.^4^, ^5^, ^7^, ^8^, ^9^, ^10^, ^11^, ^12^, ^13^, ^14^ Although these studies have provided strong support for the use of SGRT systems, most of their evidence comes from comparing the discrepancy between SGRT and the radiographic reference system's setup position and not comparing their respective dosimetric accuracies. To our knowledge, there is very little data available on the effect of SGRT setup discrepancy on a delivered dose, particularly in proton breast treatments where the setup errors could have the greatest impact.^4^, ^15^ The goal of this study was to develop a technique for replicating SGRT setup in a clinical treatment planning system and then compare the dosimetric accuracy of using SGRT‐ and cone‐beam computed tomography (CBCT)‐based setups in proton breast treatment plans. We also investigate whether there is any correlation between the dosimetric accuracy when using SGRT‐ and CBCT‐based setups and the state of the target geometry (intact vs. post‐mastectomy chest wall) and the patient's body mass index (BMI), to verify if target surface complexity and body habitus affect setup accuracy.

## 2 MATERIALS AND METHODS

### 2.1 Patient cohort

After receiving institutional review board (IRB) approval, a retrospective study was conducted on data from 30 advanced‐stage breast cancer patients (median age, 59 years; range, 27–81 years) who had been treated with pencil‐beam scanning proton therapy at our institution in 2019. Twenty‐two patients received treatment on the left breast and eight on the right; patients with bilateral breast cancer were excluded from the study. Fifteen of the patients underwent breast‐conserving surgery prior to radiation therapy, while the remaining eight and seven patients underwent mastectomy with and without reconstruction, respectively. None of the eight patients who underwent mastectomy with reconstruction had tissue expanders. The majority (20/30) of patients were treated to a dose of 5040 cGy in 28 fractions, followed by a boost of 1000 cGy in 5 fractions. Eight patients received a hypofractionated treatment regimen of 4256 cGy in 16 fractions followed by 1000 cGy in a 5‐fraction boost. The remaining two patients received 4500 cGy in 25 fractions with no boost. A summary of the patient information is provided in Supplemental Table S1.

### 2.2 Treatment planning and delivery

A free‐breathing CT simulation was performed on a Siemens Biograph scanner (Siemens Healthineers) in the head‐first supine position with the head turned away and both arms up, using an on‐arm shuttle and Vac‐Lok immobilization device. Patients were scanned from the C3 superior border to below the breast tissue in the inferior border, with a slice thickness of 3.0 mm. Three BBs were placed to mark the isocenter and wires were placed around the breast to mark the border of the breast tissue.

Contouring and treatment planning were performed in Version 15 of the Eclipse treatment planning system (Varian Medical Systems, Inc.). All treatment plans were delivered with 2 en‐face (anterior oblique, no couch kick) spot‐scanning proton beams with range shifters and were optimized to achieve 95% of the clinical target volume (CTV) receiving 100% of the prescription dose. The organs at risk (OAR) dose constraints used in planning were: heart: mean <1 Gy, V_15Gy_ <30%, V_25Gy_ <5%; ipsilateral lung: V_20Gy_ ≤20%, V_5Gy_ <40%; and esophagus: *D*_max_ <70% of prescription dose. Note that at the time the selected patients were being treated, the version of Eclipse that our clinic used for treatment planning did not yet support robust optimization techniques. Instead, those plans were non‐robustly optimized using a single field optimization (SFO) technique to optimize the proton pencil beam spot intensities of each field. To cover the CTV with the prescribed dose, a planning CTV was used during treatment optimization which covered the CTV plus a 0.5 cm margin. All the plans were then evaluated for robustness using a setup uncertainty of 5 mm and range uncertainty of 3.5%, resulting in 12 scenarios in total used for evaluation. The worst‐case scenarios were evaluated with a goal of 95% of the planning CTV being covered by 95% of prescription dose.

Treatments were delivered on a ProBeam (Varian Medical Systems, Inc.) machine with a 6‐degree‐of‐freedom robotic couch. The patient was setup with on‐board CBCT imaging and surface guidance from AlignRT (VisionRT Ltd.) for the first three fractions. Thereafter, only AlignRT was used for daily patient setup and CBCT images were acquired once per week to monitor the agreement of AlignRT with CBCT and to assess anatomical changes. The AlignRT system could only be used during patient setup when the treatment snout could be retracted and a clear line‐of‐sight to the SGRT system's cameras could be achieved.

A second CT scan, called quality assurance CT (QA‐CT), was also acquired midway through the course of treatment to keep track of anatomical changes and to assess the delivered dose accuracy. In this study, the QA‐CT dataset was used for dose calculation and was treated as a representation of the patient in the treatment position. To emulate the patient setup provided with CBCT and SGRT systems, the following registrations were performed between the QA‐CT and planning CT datasets.

### 2.3 Image registration between planning CT and QA‐CT

**FIGURE 1:**
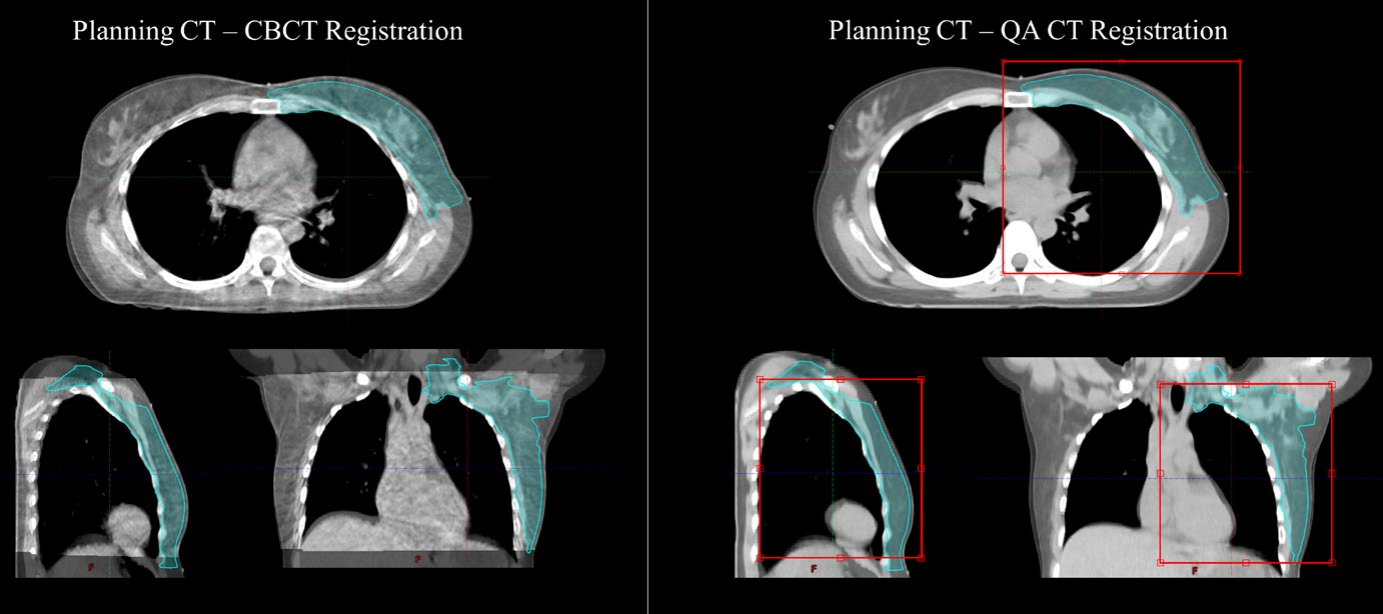
Cone‐beam computed tomography (CBCT) setup registration. Left: Orthogonal views of the planning CT and CBCT fusion. Right: Orthogonal views of the planning CT and quality assurance CT (QA‐CT) fusion with the volume used for image registration indicated by the red box. This registration volume was restricted to be within the CBCT field of view and focused on the treated volume

Two duplicates of the patient's QA‐CT image set were created for the two different types of registrations. To emulate the setup provided by CBCT imaging, we first reviewed the registration between the patient's planning CT and most recent CBCT to the QA‐CT (both were typically acquired on the same day). We then restricted the volume used for the planning CT and QA‐CT image registration to be within the superior and inferior limits of the CBCT field of view. We further reduced the anterior/posterior and left/right borders of the registration volume to the treated volumes, as this would be the region the therapist would likely focus on during the treatment setup. Finally, the planning and QA‐CT datasets were registered using this volume of interest (as shown in Figure 1) using translational and rotational shifts to achieve the best possible soft tissue and chest wall alignment between the datasets.

**FIGURE 2:**
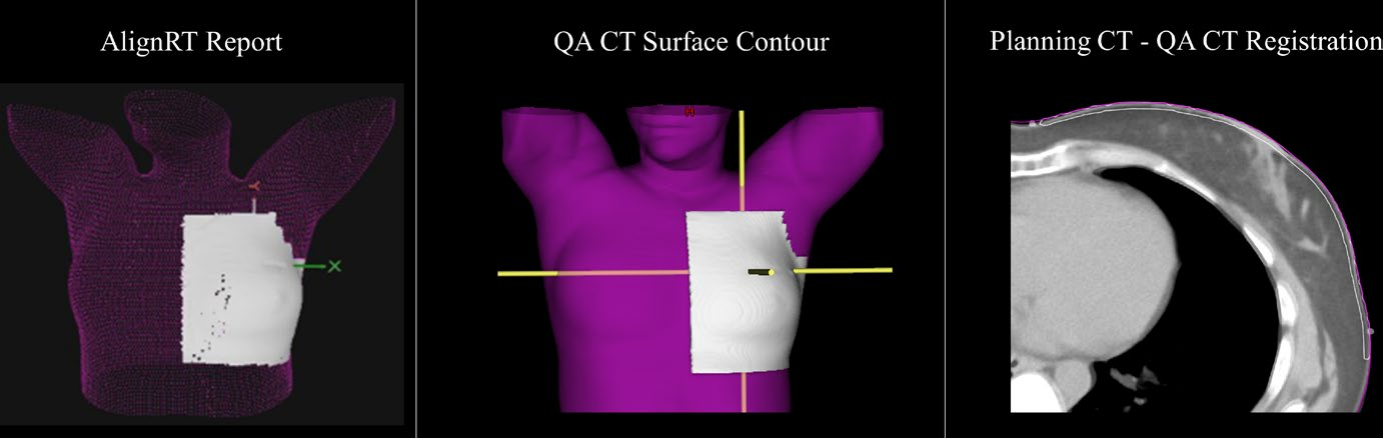
Surface‐guided radiation therapy (SGRT) setup registration. Left: The surface used for treatment setup by the AlignRT system. Middle: Recreation of the surface surveyed by the AlignRT system using a manually generated chest surface contour (shown in white) on the quality assurance CT (QA‐CT). Right: Fusion image from the planning CT and the QA‐CT after performing an automatic image registration focusing on the chest surface contour only

To emulate the setup provided by SGRT systems, we first retrieved the daily AlignRT positioning report from the patient's most recent treatment to the QA‐CT. We then created a “chest surface” contour on the QA‐CT that was a 3‐mm contraction from the surface of the breast and that was manually modified to match the 3D surface shown in the daily AlignRT positioning report (Figure 2). The planning CT and QA‐CT were then automatically registered with the registration algorithm focusing exclusively on matching the breast or chest surface contour. This resulted in good alignment between the surface of the planning CT and the QA‐CT at the breast or chest surface.

These two image registrations were then used to copy the plan and structure sets from the planning CT onto the QA‐CT. If large differences were observed between the patient anatomy in the QA‐CT and the copied structure set, the OAR contours were manually adjusted to match the patient anatomy on the QA‐CT. Manual adjustments were not made to the CTVs as wires were not placed during the QA‐CT scan to demarcate the breast tissue boundaries. Instead, Eclipse's modified‐demons deformable image registration algorithm was used to update the CTV.^16^

The dose was computed on both the QA‐CTs without disturbing the beamline. Dose metrics of the CTV (V_95%_ [%]), heart (V_25Gy_ [%], V_15Gy_ [%], mean [Gy]), and ipsilateral lung (V_20Gy_ [%], V_10Gy_ [%], V_5Gy_ [%]) were recorded for the statistical analysis. Information about target geometry (intact or post‐mastectomy without reconstruction) and their body‐mass index (BMI) was also collected to evaluate whether there was a correlation between these factors and the setup accuracy.

### 2.4 Statistical analysis

A one‐way repeated measures multivariate analysis of variance (MANOVA) was performed in the Statistical Package for the Social Sciences (SPSS Version 26; IBM Corporation) to determine whether there was a significant difference between the dose metrics calculated on the planning CT and those computed on the CBCT and SGRT‐registered QA‐CT. When the MANOVA test was significant, univariate analysis of variance was performed to determine which specific dose metrics were significantly different between the datasets. Finally, post hoc pair‐wise Student *t*‐tests were performed when the univariate tests were significant to determine which of the datasets had dose metric values that differed significantly from the others. A 5% threshold for statistical significance was used (*p* = 0.05).

## 3 RESULTS

**FIGURE 3:**
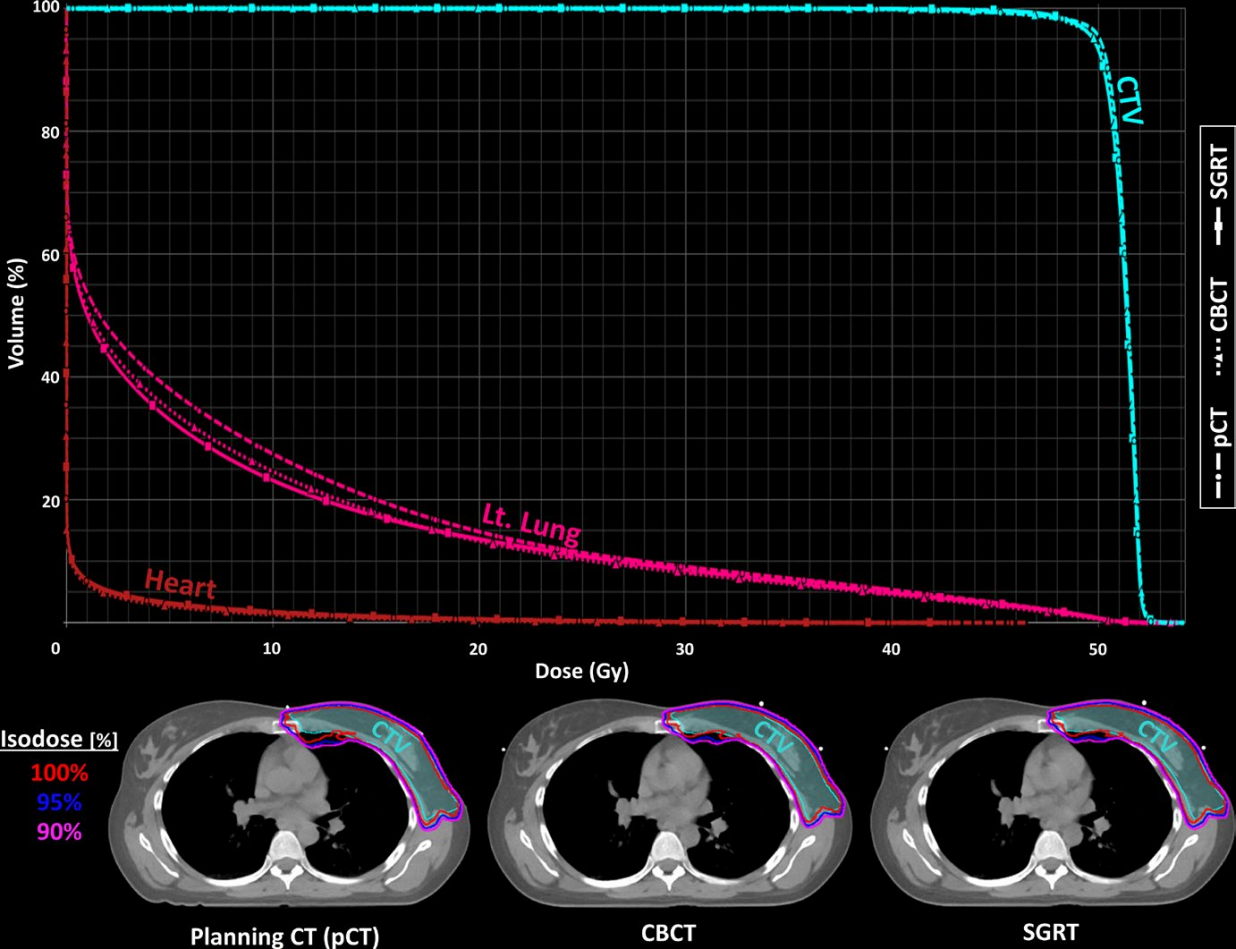
Top: Sample dose–volume histogram Bottom: Dose distributions from the planning CT (left) and the QA‐CT registered using cone‐beam computed tomography (CBCT)‐like (middle) and surface‐guided radiation therapy (SGRT)‐like (right) techniques

The average and standard deviation of each dose metric, calculated on each dataset, along with the results of the statistical analyses, are provided in Table 1. Figure 3 shows a sample dose distribution and dose–volume histogram from a representative patient. The results of the repeated measures MANOVA indicated that the dose metrics were significantly different (*p* < 0.001) between the planning CT and the QA‐CT dataset, in both the CBCT and SGRT setups. Follow‐up univariate tests found that specifically the CTV V_95%_ (*p* < 0.001) and ipsilateral lung dose metrics (*p* < 0.01 for all metrics) were significantly different between the datasets, whereas the heart dose metrics did not differ significantly. Post hoc t‐tests showed that the CTV and lung dose metrics were significantly lower in the QA‐CT than the planning CT (*p* < 0.05) in both the CBCT and SGRT setups. However, no significant difference was observed in the lung and CTV V_95%_ dose metrics between the SGRT and CBCT setups (*p* > 0.15).

**TABLE 1:** Average (standard deviation) dose metrics values over all 30 patients along with the results of the statistical analyses. *p *< 0.001 for the repeated measures MANOVA

Volume	Metric	Average (standard deviation)			ANOVA *p* value	Paired *t*‐test *p* values		
		pCT	CBCT	SGRT		pCT–CBCT	pCT–SGRT	CBCT–SGRT
CTV	V_95%_ [%]	99.2 (0.6)	97.6 (1.7)	97.8 (1.8)	<0.001	<0.001	<0.001	0.150
Heart	V_25Gy_ [%]	0.6 (1.2)	0.5 (1.3)	0.6 (1.3)	0.246	–	–	–
	V_15Gy_ [%]	1.3 (1.9)	1.1 (2.0)	1.3 (2.1)	0.332	–	–	–
	Mean [cGy]	67.1 (78.1)	59.6 (80.5)	63.8 (85.6)	0.194	–	–	–
Ipsilateral lung	V_20Gy_ [%]	10.5 (5.9)	8.5 (5.7)	8.7 (5.9)	0.010	0.005	0.022	0.162
	V_10Gy_ [%]	23.3 (10.9)	18.7 (10.3)	18.9 (10.9)	0.001	<0.001	0.001	0.676
	V_5Gy_ [%]	34.6 (13.6)	28.9 (13.4)	28.6 (14.4)	<0.001	<0.001	<0.001	0.464

Abbreviations: CBCT, cone‐beam computed tomography; pCT, planning CT; SGRT, surface‐guided radiation therapy.

No correlation was observed between the agreement of the dose metric values as a function of the patient's BMI, and so no further statistical analysis was conducted. Similarly, we observed no significant difference between the dose metrics values in the intact versus mastectomy group (*p* > 0.08 for all metrics).

## 4 DISCUSSION

In this work, we established a method of simulating CBCT‐ and SGRT‐like patient setups in a commercial treatment planning system. We then used this method to compare the dosimetric accuracy of using SGRT and image guidance with CBCT for patient setup in proton breast treatment plans. The results of this retrospective study on 30 patients are provided in Table 1.

There are three main observations from the results presented in Table 1. First, when we compare the average CTV and OAR dose metric values from the planning CT and the QA‐CT (columns 3–5 of Table 1), we observe that the dose metrics calculated on the QA‐CT are often lower than the dose metrics calculated on the planning CT, regardless as to whether a CBCT‐like setup or SGRT‐like setup is used. These differences in metrics were likely due to the differing patient anatomy and treatment positions between the planning CT and QA‐CT.

Second, the differences in the dose metrics values were only found to be significant for the CTV and lung dose metrics, while the difference in the heart dose metrics was not significant (column 6 of Table 1). Often the heart, as a whole, was far enough away from the treatment volume (as indicated by the low heart mean and V_25Gy_ and V_15Gy_ values in Table 1) that any difference in anatomy between the planning CT and QA‐CT as well as any difference in setup position had very little impact on the dose that this OAR received. That said, the left anterior descending (LAD) artery of the heart might have received considerably higher doses being closer to the treated volume. Unfortunately, the patients in this study did not have their LAD arteries delineated and, as a result, the LAD dose was not analyzed in this study.

Finally, when we compared between the dose metrics obtained in the CBCT and SGRT‐like setup position, we observed no significant difference in the lung and CTV V_95%_ dose metrics. This indicates that the SGRT‐setups provided similar dosimetric accuracy to the CBCT‐based setup. Given that SGRT systems are often faster and easier to use than CBCT and do not require imaging dose, SGRT will be beneficial over CBCT for the daily setup of proton breast treatment plans. In our center, CBCT‐based setups take around 5 minutes longer than the SGRT based setups, hence with the SGRT setup, patients may spend 5 minutes less on the table. That said, the accuracy of SGRT should still be verified on a patient‐by‐patient basis during the first few fractions of treatment using kV‐pairs or CBCT. Furthermore, routine CBCT or QA‐CT should still be acquired to verify anatomical consistency and dosimetric accuracy throughout the treatment course.

We also investigated whether other patient factors such as body mass index (BMI) or target state (intact or post‐mastectomy without reconstruction) had an impact on the setup accuracy of SGRT systems. The target state had no detectable impact on the agreement between the planning CT and QA‐CT dose metric values when using either the CBCT or SGRT setup. Similarly, we observed no correlation between the agreement of the dose metric values and the patients’ BMI. Since there was no clear relationship between the agreement of the dose metric values and these factors, no multiple regression analysis was performed.

The method used in this work could be used to evaluate the dosimetric accuracy of using SGRT systems in other treatment scenarios, such as deep inspiration breath‐hold in proton breast treatments and frameless stereotactic body radiotherapy. Those topics will be investigated in a future study.

## 5 CONCLUSIONS

The results of this study support the use of SGRT systems for patient setup of intensity‐modulated proton therapy breast treatment plans. SGRT provided comparable dosimetric accuracy to that of image guidance with CBCT.

## CONFLICT OF INTEREST

None.

## AUTHOR CONTRIBUTIONS

All listed authors contributed to the study and to drafting the manuscript.

## Supporting information

Table S1
