# Supplementary material for: Comparison of the dosimetric accuracy of proton breast treatment plans delivered with SGRT and CBCT setups
Source: J Appl Clin Med Phys. 2021 Jul 20;22(9):153–8. doi: 10.1002/acm2.13357 (PMC8425866; doi:10.1002/acm2.13357)
Supplement: Supplementary file 1 — Table S1 [file ACM2-22-153-s001.docx]

| **No.** | **Age (Y)** | **Lt/Rt Breast** | **Dose (cGy)** | **No. of Fractions** | **Status post** | **CNI** | **CTV Vol. (cc)** | **Heart Vol. (cc)** | **Ipsilateral Lung Vol. (cc)** |
| --- | --- | --- | --- | --- | --- | --- | --- | --- | --- |
| 1 | 70 | R | 5040 | 28 | Lumpectomy | Y | 2445.5 | 672 | 1192.6 |
| 2 | 65 | L | 5040 | 28 | Lumpectomy | Y | 3042.8 | 794 | 897 |
| 3 | 58 | R | 5040 | 28 | Lumpectomy | Y | 1104 | 734.3 | 1591.3 |
| 4 | 67 | R | 5040 | 28 | Lumpectomy | Y | 1228.8 | 431.1 | 1210.4 |
| 5 | 27 | L | 5040 | 28 | Mastectomy + Implant | Y | 1715.2 | 674.7 | 700.5 |
| 6 | 81 | L | 5040 | 28 | Mastectomy w/o reconstruction | IMN only | 956.3 | 500.2 | 907.6 |
| 7 | 67 | L | 5040 | 28 | Mastectomy + Implant | Y | 2133.5 | 551.2 | 575.9 |
| 8 | 49 | L | 4256 | 16 | Lumpectomy | N | 1696.2 | 660.6 | 822.1 |
| 9 | 67 | R | 5040 | 28 | Mastectomy w/o reconstruction | Y | 1435.2 | 746.7 | 1133.1 |
| 10 | 38 | L | 5040 | 28 | Lumpectomy | Y | 932.8 | 526.8 | 1272.2 |
| 11 | 52 | L | 4256 | 16 | Lumpectomy (pt. w implants) | N | 1545.1 | 674.4 | 1259.7 |
| 12 | 51 | L | 5040 | 28 | Mastectomy w/o reconstruction | Y | 1066.3 | 582.1 | 871.3 |
| 13 | 67 | L | 5040 | 28 | Mastectomy w/o reconstruction | Y | 652.3 | 838.7 | 1844.3 |
| 14 | 59 | R | 5040 | 28 | Mastectomy w/o reconstruction | Y | 727.4 | 608.5 | 1425.2 |
| 15 | 70 | L | 4500 | 25 | Mastectomy w/o reconstruction | N | 158.2 | 605.2 | 905.5 |
| 16 | 74 | L | 5040 | 28 | Lumpectomy | Y | 1516.6 | 517.7 | 1060.6 |
| 17 | 58 | L | 4256 | 16 | Lumpectomy | N | 658 | 512.4 | 1333.5 |
| 18 | 41 | L | 5040 | 28 | Mastectomy w/o reconstruction | Y | 1096.1 | 654.8 | 1192 |
| 19 | 48 | R | 5040 | 28 | Mastectomy + Implant | Y | 1023 | 680.5 | 1420.5 |
| 20 | 80 | L | 4256 | 16 | Lumpectomy | N | 1093.5 | 698.8 | 841.8 |
| 21 | 67 | L | 4256 | 16 | Lumpectomy | N | 1631 | 674.5 | 1012.4 |
| 22 | 48 | R | 4256 | 16 | Lumpectomy | N | 1841.3 | 703.2 | 1280.8 |
| 23 | 39 | L | 5040 | 28 | Mastectomy w implant | Y | 1127.2 | 557.2 | 1203.1 |
| 24 | 30 | L | 4500 | 25 | Mastectomy w reconstruction | Y | 1351.3 | 554.3 | 871.7 |
| 25 | 34 | L | 5040 | 28 | Mastectomy w reconstruction | Y | 906 | 468.4 | 1084 |
| 26 | 65 | L | 5040 | 28 | Lumpectomy | Y | 2230 | 650.2 | 692.3 |
| 27 | 50 | R | 5040 | 28 | Mastectomy w reconstruction | Y | 1357.5 | 704 | 1193.33 |
| 28 | 50 | L | 5040 | 28 | Mastectomy w reconstruction | Y | 755 | 443.1 | 968.4 |
| 29 | 69 | L | 4256 | 16 | Lumpectomy | N | 750.7 | 668.2 | 1108.2 |
| 30 | 75 | L | 4256 | 16 | Lumpectomy | N | 420.7 | 695 | 1008.7 |
